# Southern Hemisphere forcing of South Asian monsoon precipitation over the past ~1 million years

**DOI:** 10.1038/s41467-018-07076-2

**Published:** 2018-11-08

**Authors:** D. Gebregiorgis, E. C. Hathorne, L. Giosan, S. Clemens, D. Nürnberg, M. Frank

**Affiliations:** 1 GEOMAR Helmholtz Centre for Ocean Research Kiel, Wischhofstraße 1-3, 24148 Kiel, Germany; 2 Department of Geosciences, Georgia State University, Atlanta, GA 30303 USA; 3 Woods Hole Oceanographic Institution, Woods Hole, MA 02543 USA; 4 Earth, Environmental, and Planetary Sciences, Brown University, Providence, RI 02912 USA

## Abstract

The orbital-scale timing of South Asian monsoon (SAM) precipitation is poorly understood. Here we present new SST and seawater δ^18^O (δ^18^Osw) records from the Bay of Bengal, the core convective region of the South Asian monsoon, over the past 1 million years. Our records reveal that SAM precipitation peaked in the precession band ~9 kyrs after Northern Hemisphere summer insolation maxima, in phase with records of SAM winds in the Arabian Sea and eastern Indian Ocean. Precession-band variance, however, accounts for ~30% of the total variance of SAM precipitation while it was either absent or dominant in records of the East Asian monsoon (EAM). This and the observation that SAM precipitation was phase locked with obliquity minima and was sensitive to Southern Hemisphere warming provides clear evidence that SAM and EAM precipitation responded differently to orbital forcing and highlights the importance of internal processes forcing monsoon variability.

## Abstract (web summary)

The orbital-scale timing of South Asian monsoon precipitation is poorly understood. Here the authors show that the long held view that precession drove changes in monsoon strength is wrong, and that obliquity and eccentricity played a stronger role.

## Introduction

By 2050 over 5 billion people will live in the region directly influenced by the South Asian monsoon (SAM)^1^, for whom a better understanding of the forcing mechanisms and improved forecasts will be critical. The conventional view is that the SAM is primarily driven by interhemispheric temperature/pressure gradients between the Asian continent (particularly low pressure zones over the Tibetan Plateau and India) and the southern subtropical Indian Ocean^2^, although the role of Tibetan plateau summer warming has recently been challenged^3^. Variability in SAM precipitation over the instrumental period has been considered to be directly linked to ENSO in that monsoonal precipitation over land tends to decrease during El Niño years^4^. However, under the influence of global warming this relationship has become markedly less clear^5,6^. It is thus not surprising that simulating the basic aspects of the SAM remains difficult for even the most advanced coupled ocean–atmosphere general circulation models^7^. The same holds for our understanding of the link between monsoon precipitation and Northern Hemisphere (NH) summer insolation on orbital time scales, where insolation is modulated by Earth’s precession, obliquity and eccentricity cycles^8^.

Previous studies of SAM variability on orbital time scales have mainly relied on wind-based proxies from the Arabian Sea^9,10^. Interpretation of the biological proxies is based on modern observations demonstrating that strong summer monsoon winds promote upwelling-driven productivity and a distinct plankton assemblage^11^. Lithogenic grain size proxies are directly linked to the transport capacity of the summer monsoon winds^12^. In the eastern Indian Ocean, Bolton et al.^13^ used δ^18^O gradients between two planktic foraminiferal species to reconstruct wind-driven upper ocean stratification changes which, unsurprisingly, exhibit orbital frequencies similar to the wind-driven proxies from the Arabian Sea. Several of these records indicate a consistent time lag of ~8–9 kyrs between peak NH summer insolation and peak wind intensity at the precession band^9,10^. This is in direct contrast to the speleothem δ^18^O records from central China that reflect the isotopic composition of East Asian monsoon (EAM) precipitation^14^ and lag NH insolation by only ~3 kyrs^15–18^ in the precession band. The trans-regional complexity of the monsoon response to orbital forcing is further demonstrated by a speleothem δ^18^O record from Southwest China that is directly in-phase with precession^19^. The fact that the δ^18^O signal of precipitation does not directly reflect rainfall amount^14^ implies that records integrating the signal from large river catchments are more likely to reflect monsoon rainfall. A new δ^18^Osw record integrating the signal from the Yangtze river valley indicates that precession and direct insolation forcing were not the dominant drivers of the EAM^20^. This highlights a fundamental lack of understanding of the internal climate processes regulating the Asian monsoon response to insolation forcing. This situation is perpetuated by the lack of proxy records of monsoon precipitation variability rather than that of wind. The assumption that monsoon rains and winds have been linearly related in the past has yet to be tested and, given that it is the amount and intensity of monsoon precipitation which are of direct societal relevance, filling this knowledge gap is vitally important. The ideal location for recovering such records is the core convective region of the South Asian monsoon in the Bay of Bengal where the existing short monsoon precipitation record^21^ can now be extended using recently obtained drill cores.

**Fig. 1:**
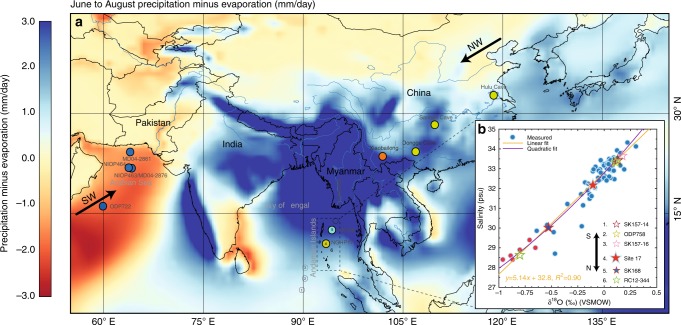
Summer net precipitation in the Asian monsoon domain and modern day salinity and δ^18^Osw measurements. **a** Precipitation minus evaporation (mm/day) in the Asian monsoon domain for the period 1979–2015 (precipitation and evaporation data from ERA-Interim global reanalysis dataset). Andaman Sea core sites NGHP 17 (ref. ^27^) and SSK 168 (refs. ^28,29^) are shown in yellow and dark green-filled circles. Filled circles show cave and core locations from mainland China^15,19^, the Arabian Sea^9,10^ and the equatorial eastern Indian Ocean (ODP758)^13^. **b** Paired δ^18^Osw and salinity measurements from surface water samples collected from the Andaman Islands in 2011 and 2013 (see Methods) and estimates of δ^18^Osw calculated based on planktic foramifera late Holocene (0–2 kyrs) δ^18^O values and modern day mean annual SST^50^(see Methods for δ^18^O–temperature calibration equation) for six core locations along the N-S transect shown in black circles with numbers (see legend). Measured δ^18^Osw values shown in red-filled circles are from Achyuthan et al.^51^ and are collected close to the Andaman Islands in December 2008

The Bay of Bengal has a low-salinity surface “boundary layer”, that is present year round, caused by river runoff and direct precipitation over the ocean (Fig. 1). The net annual surface freshwater budget (i.e. precipitation plus runoff minus evaporation) is primarily driven by intense freshening of the surface ocean during the summer monsoon season (see Supplementary Note 1 for detailed description of the regional oceanography) as confirmed by in situ observations of salinity in the Bay of Bengal and the Andaman Sea^22,23^. Sea surface salinity (SSS) variability in the Andaman Sea and Bay of Bengal is characterised by a semi-annual cycle of mixed layer and thermocline depth variations intrinsically linked to the seasonally reversing monsoonal circulation^24,25^. Some of this is the result of wind-driven mixing but the amount of freshwater in the boundary layer is ultimately controlled by monsoon precipitation in preceding years.

Here, we combine Mg/Ca measurements (a proxy for the temperature prevailing during calcification) and oxygen isotope (δ^18^O) analyses of the calcite shells of mixed-layer dwelling planktic foraminifera *Globigerinoides sacculifer* (present all year round in the Bay of Bengal^26^) in core NGHP 17 (ref. ^27^) to generate a unique orbital scale SAM precipitation record for the last **~**1 million years. Furthermore, we replicated these findings with a deeper dwelling species *Neogloboquadrina dutertrei* known to thrive under the summer monsoon conditions near the fresh boundary layer in the Bay of Bengal^26^.

Our results reveal that SAM precipitation was weak during glacial maxima and generally stronger during interglacials. Superimposed on this, higher frequency variations of SAM precipitation peaked in the precession band ~9 kyrs after NH insolation maxima, in-phase with wind-driven changes in the Arabian Sea^9,10^ and upper ocean stratification in the Eastern Indian Ocean^13^. In contrast to the precession-dominated wind-driven records and the speleothem records from the EAM domain^15–19^, the precession band only accounts for ~30% of the total variance of SAM precipitation and thus cannot be considered the primary driving force. At the same time, the fact that precession-driven variability is missing in new δ^18^Osw records from the East China Sea^20^ provides the clearest evidence that the East Asian and South Asian monsoon systems have responded independently to orbital insolation changes. Our SAM precipitation record demonstrates that obliquity forcing has played a much larger role than previously considered and was triggered by Southern Hemisphere warming and cross hemispheric moisture transport rather than NH insolation.

## Results

### δ^18^O and salinity relationship

The δ^18^O signature of planktic foraminifera is a function of calcification temperature and the δ^18^O of ambient seawater (δ^18^Osw). The close relationship between salinity and the oxygen isotope (δ^18^O) composition of seawater in the region is verified by our new paired δ^18^Osw, and salinity measurements of surface water samples collected from the Andaman Islands in 2011 and 2013 (Fig. 1). The salinity and δ^18^Osw gradient across the Bay of Bengal is replicated by the δ^18^O composition of planktic foraminifera from core sites across the Bay of Bengal confirming the fidelity of this proxy^28,29^ (Fig. 1). The Mg/Ca ratio of planktic foraminiferal calcite has the unique advantage of being an independent temperature proxy that is measured on the very same shells as δ^18^O. Paired Mg/Ca–δ^18^O measurements therefore allow the accurate reconstruction of temperature and δ^18^Osw signals. Given the regional nature of salinity–δ^18^Osw relationships, we cannot extrapolate the modern relationship to provide absolute SSS values for the past (especially during drier intervals). However, the reconstructed changes in δ^18^Osw are principally a function of the amount of fresh surface water in the boundary layer and proportional to SSS changes in the Andaman Sea. This provides a unique, continuous summer monsoon precipitation intensity record from the heart of the SAM core convective region.

### SST and δ^18^Osw records

**Fig. 2:**
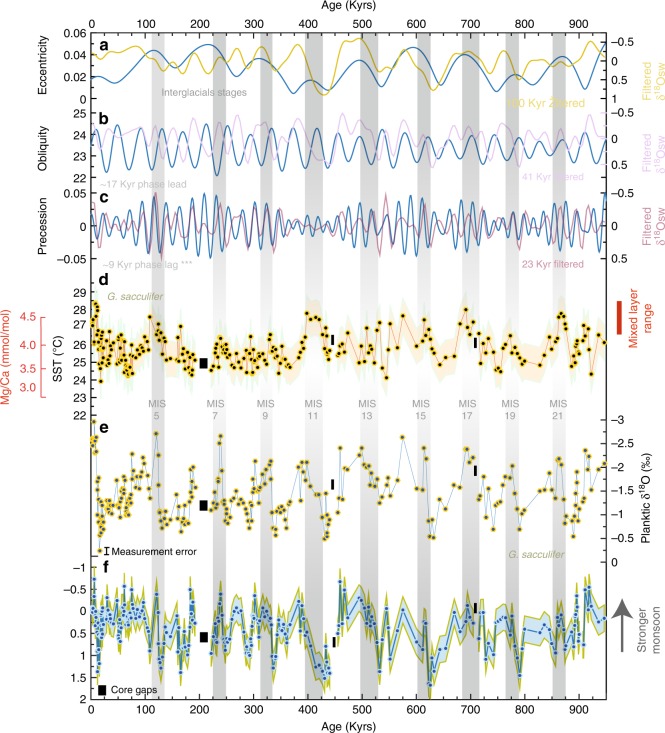
Proxy timeseries. **a** Comparison of 100 kyrs eccentricity^30^ (blue) and 100 kyrs filtered δ^18^Osw. **b** Comparison of 41 kyr obliquity^30^ (blue) and 41 kyrs filtered δ^18^Osw. **c** Comparison of 23 kyr precession^30^ (blue) and 23 kyrs filtered δ^18^Osw. **d** Reconstructed SST at site 17. **e** Oxygen isotope ratios of planktic foraminifer *G. sacculifer*. **f** Ice volume corrected δ^18^Osw. Envelopes in **d** and **f** denote uncertainties in δ^18^Osw and SST records estimated by propagating errors introduced by the δ^18^O and Mg/Ca measurements and calibration equations, and are on average ~1 °C and ~0.3‰, respectively. Bars show major interglacial periods during the last ~1 Myrs. Also shown are δ^18^Osw phase lags with respect to the three main orbital frequencies (***denotes 95% CI)

**Fig. 3:**
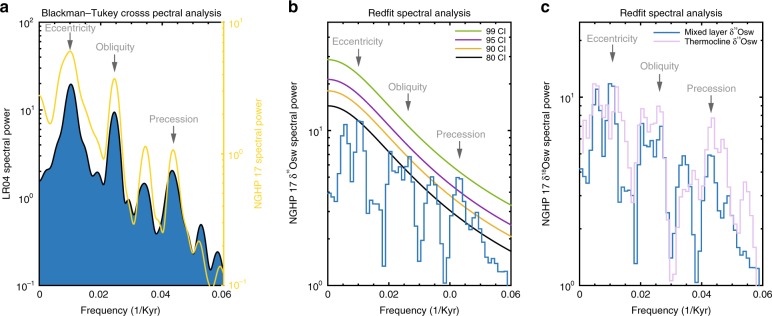
Cross spectral and spectral analysis of proxy timeseries. **a** Blackman–Tukey cross spectral analysis of LR04 (Ref.^35^) and NGHP 17 benthic oxygen isotope record indicating strong coherency at the three main orbital frequencies (95% CI). **b** Redfit Spectral analysis^46^ of ice volume corrected δ^18^Osw record. **c** Redfit Spectral analysis^46^ of Ice volume corrected δ^18^Osw and thermocline δ^18^Osw records showing remarkably consistent cycles. Spectral analyses were performed on unevenly spaced time series with an oversampling factor of two and a Hanning window to define spectral peaks

The reconstructed SST, δ^18^O and δ^18^Osw show consistent patterns for the past ten glacial–interglacial periods documenting generally weak monsoon intensity during glacial maxima, in agreement with previous reconstructions for the last glacial maximum^28,29^, and a strong monsoon during the past ten interglacials (Fig. 2). Superimposed on this pattern are higher frequency variations driven by precession and obliquity, indicating relatively strong intervals of monsoon precipitation within glacial stages 4 and 6. Coldest SSTs between 24 and 25 °C are observed consistently during all major glacial stages (e.g. LGM, MIS 4, MIS 6 and MIS 10) while the onsets of MIS 11, MIS 17 and MIS 21 were marked by significantly warmer SSTs (~28 °C). This is also clearly reflected in the spectral analysis with SST, δ^18^O, δ^18^Osw dominated by ~100 kyr eccentricity, 41 kyr obliquity (tilt) and ~23 kyr precession (ETP)^30^ cycles (>90% CI) (Fig. 3). One key point to note is that δ^18^Osw variability within the mixed layer and thermocline in the Andaman Sea are remarkably consistent with each other (see Fig. 3c and Supplementary Note 1). SST and δ^18^Osw variations are highly coherent with precession (>95% CI) and to lesser extent in the obliquity band (>80% CI). Precession-related variance in the δ^18^Osw records, however, accounts for at most 30% of the total variance.

### Phasing of South Asian monsoon precipitation

**Fig. 4:**
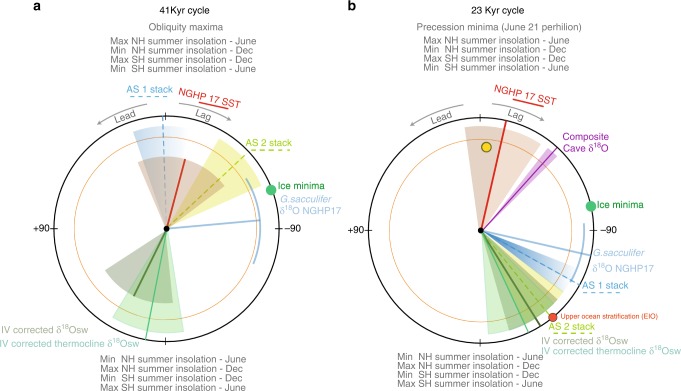
Phase wheels illustrating Asian monsoon response to orbital forcing. **a** Obliquity (41 kyr) and **b** precession (23 kyr) phasing of Asian monsoon proxy records during the late Quaternary. The precession index is defined as Δ*ε*sin*ω*, where *ω* is the longitude of the perihelion measured from the moving vernal point and *ε* is the eccentricity of Earth’s orbit around the Sun^52,53^. Obliquity is the axial tilt of Earth’s rotation axis with respect to the orbital plane. In the phase wheel representation, the 12 o’clock position is set to precession minima (*P* min; *ω* = 90°, 21st June perihelion) and obliquity maxima (*O* max), respectively. Negative/positive phases are measured in the clockwise/anticlockwise direction and represent phase lags/leads relative to *P* min or *O* max. Published records plotted (with shaded phase estimate errors) are the Arabian Sea summer monsoon stacks AS1 (ref. ^10^) and AS2 (ref. ^9^), composite cave δ^18^O records from southwest^15^ (shown in yellow circle) and east China^19^, upper ocean stratification record from the eastern Indian Ocean (EIO)^13^ and IV-corrected δ^18^Osw records from NGHP 17 (this study). Orange circle denotes 80% coherency The 95% and 80% confidence intervals for coherency are 0.79 and 0.64 with bandwidth 0.00423175. The interpolated time interval is 3.5 kyrs and covers 3 to 951 kyrs. Phase errors for the IV-corrected mixed layer and thermocline δ^18^Osw records at the precession band are ± 17° and ± 20°, respectively. Phase errors for the IV-corrected mixed layer and thermocline δ^18^Osw records at the obliquity band are ± 36° and ± 20°, respectively

To understand the link between tropical SST in the Andaman Sea and monsoon precipitation, we analysed phase (time lead/lag) relationships between SST and δ^18^Osw records with respect to ETP^30^. In addition, we compared the phasing with wind records (two Arabian Sea monsoon stacks hereafter denoted AS1^9^ and AS2^10^, and upper ocean stratification in the equatorial eastern Indian Ocean^13^), and the recently published composite cave δ^18^O records from Southwest^19^ and East China^15^ to understand the variability of the Asian monsoon systems on orbital time scales. Figure 4 summarizes coherence and phase relationships between our new Andaman Sea records and ETP^30^ in the precession and obliquity bands. Only precession and obliquity phase wheels are presented as the very small eccentricity changes trigger only negligible ~100 kyr power in seasonal or mean annual insolation variations and cannot account for the dominant ~100 kyr cyclicity. The SST record strictly follows peak insolation changes without delays. In stark contrast, the δ^18^Osw derived from the same samples significantly lags maximum NH insolation by ~9 kyrs in the precession band (Fig. 4). This is also consistent with the ~4 kyrs phase lag of the δ^18^Osw record with respect to ice volume minima inferred from the benthic δ^18^O record (see methods). In the obliquity band, the Andaman Sea δ^18^Osw records are nearly in-phase with obliquity minima. The δ^18^Osw lag appears to be close to ice volume maxima and sensible heat minima, which lead/lag obliquity maxima/minima by ~14 and 6 kyrs, respectively.

## Discussion

Our new SAM precipitation record has the same precession-band phasing as the SAM wind-strength/upwelling records; all are offset by ~3 to ~5 kyrs with respect to minimum ice volume and the Chinese cave δ^18^O records^15^. This ~9 kyr lag between Andaman Sea SST and δ^18^Osw records demonstrates that direct NH summer insolation forcing has not set the timing and strength of SAM precipitation in the precession band. At the same time, nearly identical phasing between the thermocline δ^18^Osw and mixed layer δ^18^Osw (see Fig 4 and Supplementary Figure 10) records substantiate that the strong monsoon precipitation signal has consistently been imprinted within the relatively fresh layer extending down to the thermocline. The strong coupling between basin-scale monsoon winds over the Arabian Sea and eastern Indian Ocean and SAM precipitation, and the significantly longer phasing offset with respect to minimum ice volume supports the role of Southern Hemisphere warming in triggering the cross-equatorial transport of latent heat powering the SAM circulation^10,12^.

Our results indicate broad-scale precession-band coherence within the SAM region, from the Arabian Sea through to the Bay of Bengal, as derived from a wide variety of monsoon proxies. These consistent results argue against the hypothesis that tropical monsoon (EAM and SAM) variability is dominated by and responds directly to NH summer solar radiation on orbital time scales, a hypothesis strongly debated since it was promoted by Wang et al.^31^The new δ^18^Osw records from the East China Sea off the Yangtze river do not show the precession-related variability that dominates the speleothem records from Yangtze river valley caves, suggesting that EAM precipitation is more sensitive to internal forcing such as greenhouse gas and high-latitude ice sheet dynamics rather than directly responding to NH summer insolation^20^. It is not surprising that a record of upper ocean stratification from the equatorial eastern Indian Ocean^13^ exhibits orbital frequencies similar to the wind-based proxies from the Arabian Sea given that the equatorial region is well beyond the influence of monsoon freshwater (Fig. 1). The wind forced records from the Arabian Sea and Eastern Indian Ocean^13^ are dominated by orbital precession, while our new δ^18^O_sw_ record of SAM precipitation is clearly distinct from both the SAM wind proxies and the new EAM precipitation records^20^. This is indicative of complex internal forcing and demonstrates the spatial heterogeneity of monsoon precipitation response to orbital forcing over the vast Asian continent, as well as the decoupling of SAM precipitation from wind intensity and stratification changes over the Arabian Sea and eastern Indian Ocean. In particular, the prevalence of distinct spectral peaks in the new SAM δ^18^Osw record at ~178 kyrs and ~30 kyrs, which represent heterodynes of the primary orbital periods demonstrates that the response of SAM precipitation to orbital forcing, similar to EAM precipitation^20^, was strongly non-linear. SAM precipitation was clearly sensitive to internal processes that combine precession, obliquity and eccentricity band forcing documenting that precession forcing was not the sole driver of SAM precipitation.

In the obliquity band, the Andaman Sea δ^18^Osw records are nearly in-phase with obliquity minima. This is opposite to obliquity timing estimates of summer monsoon wind maxima in the Arabian Sea^9,10^, while records of upper ocean stratification from the Eastern Indian Ocean^13^ only show weak coherence with obliquity and negligible obliquity related variance. This demonstrates that SAM precipitation is mainly driven by different processes from those affecting SAM wind intensity, and is more sensitive to obliquity driven warming in the Southern Hemisphere high latitudes. The δ^18^Osw lag appears to be close to ice volume maxima and sensible heat minima, which lead/lag obliquity maxima/minima by ~14 and 6 kyrs, respectively. This nearly 180° out-of-phase relationship between monsoon precipitation and maximum obliquity indicates that neither decreased ice volume nor increased latent heat export following obliquity maxima set the phasing of SAM precipitation. We instead propose that obliquity forcing has played a vital role in local monsoon intensification through enhanced cross-hemispheric atmospheric moisture fluxes given that the reduction in Earth’s obliquity forcing induce strong summer thermal gradients between the two hemispheres^32^. The observed monsoon intensification during periods of decreased obliquity forcing and some glacial stages (e.g. Fig. 2. MIS 4 and 6) is thus likely due to the net poleward moisture transport that was triggered by enhanced heat transport following Southern Hemisphere warming. In addition, asymmetric heating of the tropics following decreased obliquity may have led to a La Niña like state and a strengthening of the Walker circulation, which would have brought low pressure zones closer to the South Asian subcontinent^33^. On the other hand, the presence of the ~100 kyr cycle in these records (Fig. 3) indicates that sea-level changes may have also influenced basin isolation and monsoon intensification.

Our new records provide compelling evidence that precession-driven variability of SAM precipitation around the Andaman Sea is consistent with that observed in SAM wind/upwelling and upper ocean stratification proxies from the Arabian Sea and eastern Indian Ocean, supporting a strong sensitivity to Southern Hemisphere (SH) warming. However, in contrast to these records we show that obliquity plays a vital role in the orbital pacing of SAM precipitation via combined SH warming and asymmetric heating of the tropics. In the course of global warming of the last century, the wind speed over the Arabian Sea has increased while precipitation over India exhibited no clear trend^34^. This decoupling is similar to that observed between maximum obliquity forcing of past SAM winds and records of upper ocean stratification in the Eastern Indian Ocean^13^. In view of these results, the role of atmospheric moisture flux triggered by hemispheric insolation gradients and the feedback processes between ENSO and Southern Hemisphere climate need to be examined using transient model simulations in the future. A better understanding of these mechanisms will significantly improve the prediction of SAM precipitation.

## Methods

### Age model and oxygen isotope stratigraphy of NGHP 17

In 2006, the Indian National Gas Hydrate Program (NGHP) used the IODP vessel JOIDES Resolution to core Site 17 (10° 45.19’ N, 93° 6.74’ E) in a water depth of 1356 m in the Andaman Sea^27^. The upper 118 m of foraminifera-rich nannofossil ooze was APC cored with excellent recovery, and we present data here for the upper ~50 m of the core. The age model of site 17 was constructed by aligning unambiguous glacial–interglacial ~100 kyr cycles of the benthic δ^18^O record with an amplitude of 1.75 ‰, with equivalent features in the LR04 global benthic δ^18^O stack^35^ using Analyseries^36^ (Supplementary Figure 1). *C. wuellerstorfi* and *C. mundulus* are epibenthic foraminiferal species, and δ^18^O values were adjusted to equilibrium by adding 0.64 following Shackleton et al.^37^. For the upper section of the core, the age model was further supported by five ^14^C dates converted to conventional ages and the identification of the youngest Toba ash layer^38^. The AMS ^14^C ages were corrected for marine reservoir ages that were determined locally^39^. Core scanning XRF Ti/Ca ratios from this core and a recently drilled nearby core (U1448) were used to determine the length of the inconsequential core gaps at Site NGHP 17 (Supplementary Figure 2). The mean sedimentation rate remained relatively constant throughout the studied period and is on average 5 cm/kyr (Fig. 1b). To investigate whether this tuning approach is robust, we performed spectral and evolutionary spectra wavelet analysis of the resulting NGHP 17 benthic record in both age and depth domains (Supplementary Figure 3). Spectral analysis of the NGHP 17 benthic record in the depth domain reveals strong power spectra at 0.002, 0.005 and 0.007 m/cycle and to a lesser extent at 0.003 and 0.006 m/cycle. These periodicities have nearly precise 1:1.5:4 frequency ratios, which demonstrate the presence of Milankovitch-related periodicities in the depth domain coherent with the age domain. The absence of a strong shift in the depth domain wave bands also confirms that sedimentation rates remained largely constant. The accuracy of the age model depends on the accuracy of the assumption of synchronicity with the LR04 benthic stack^35^. During the period with radiometric age constraints and the Toba ash this assumption seems valid (Supplementary Figure 1). Consequently, phasing offsets between NGHP 17 benthic record and the LR04 benthic stack^35^ are used to quantify age model uncertainties. The phase error associated with our age model excluding the uncertainty associated with the benthic stack^35^ is ± 3 kyrs for the 100 kyrs-eccentricity cycle, ± 0.5 kyrs for the 41 kyrs-obliquity cycle and ± 1 kyrs for the 23 kyrs-precession cycle (Supplementary Figure 4). We have performed a phase analysis entirely internal to NGHP 17– i.e. the phase of δ^18^Osw record with respect to the benthic δ^18^O record (two parameters measured on the same samples). In this case, tuning was not performed and the results are summarized in Supplementary Figure 5, where coherence and phase relationships between δ^18^Osw and benthic δ^18^O records are presented for the precession and obliquity bands. Consistent with our LR04-based age model, the δ^18^Osw record shows a significant lag of ~4 kyrs with respect to ice volume minima inferred from the benthic δ^18^O record (Supplementary Figure 5). This confirms that phasing offsets between the δ^18^Osw record and ETP^30^ are not artefacts of the age model but rather robust signals driven by internal processes of the climate system.

### SST and δ^18^Osw reconstructions

More than 50 individual *Globigerinoides sacculifer* (without sack like final chamber) or *Neogloboquadrina dutertrei* shells selected from the 315–400-μm size fraction were cracked before being split for stable isotope and Mg/Ca analyses. Foraminiferal stable isotope analysis was performed at GEOMAR using a MAT 253 mass spectrometer coupled with a Kiel IV Carbonate device system (Thermo Scientific). Mg/Ca ratios were measured with an Agilent 7500*cs* ICP-MS (for samples up to 22 m depth) and ICP OES (VARIAN 720–ES) (for samples below 22 m depth). Precision estimates for Mg/Ca are based on replicate measurements of ECRM 752-1 limestone reference material and are on average ± 0.06 mmol/mol (1σ) for both instruments. The average Mg/Ca of ECRM 752-1 measured during the course of the study was 3.8 ± 0.06 mmol/mol, which agrees well with the consensus value (ref. ^40^). For details of the Mg/Ca cleaning followed, see ref. ^28^. To exclude biases due to possible contamination, we have carefully monitored Al/Ca, Fe/Ca and Mn/Ca ratios^41^. For the dataset presented here no systematic relationships exist between Al/Ca (or Fe/Ca) and Mg/Ca ratios and we exclude the possibility of clay contamination. However, Al/Ca values in 16 samples were significantly higher and these data were excluded from further interpretations. SSTs were calculated by using the multispecies equation of Anand et al.^42^ Seawater δ^18^O (δ^18^Osw) was calculated using the δ^18^O–temperature calibration of Bemis et al.^43^ The δ^18^Osw record was corrected for global ice volume following Rohling et al.^44^ based on the chronology of the LR04 global benthic δ^18^O stack^35^ and results are similar to ice volume correction following Waelbroeck et al.^45^ (Supplementary Figure 6). δ^18^Osw values were converted to Vienna Standard Mean Ocean Water (VSMOW) by adding 0.27‰. Uncertainties in δ^18^Osw and SST were estimated by propagating maximum possible analytical errors of the δ^18^O and Mg/Ca measurements, calibration equations for both temperature and the δ^18^Osw, and the global ice volume corrections ( ± 0.09‰) (see Supplementary Note 2). The possible influence of salinity on Mg/Ca temperature estimates was investigated and found to not influence the results significantly (see Supplementary Figure 11). Spectral analyses were performed on unevenly distributed time series using REDFIT^46^. Coherence and phase analysis were performed using the Arand software package^47^.

### Modern salinity measurements

Seawater samples were collected from a wooden boat or while snorkelling around the Andaman Islands in 2011 and 2013 in acid pre-cleaned 1 litre PE bottles rinsed with 18.2 MΩ water. Samples were taken with minimal air space and were filtered on the day of collection through 0.2 micron cellulose nitrate membrane filters. Directly after filtering, 1.5 mL glass vials were filled with sample, so no air bubbles were present, and the lid with septum was sealed using parafilm. Samples were analysed for δ^18^O and δ^2^H by isotope ratio infrared spectroscopy (L 1102-*i* WS-CRDS, Picarro Inc., Santa Clara, CA, USA) at the Friedrich-Alexander University Erlangen-Nürnberg, Germany. All values are reported in the standard δ-notation (‰) versus VSMOW and external reproducibility based on repeated analyses of a control sample was better than 0.1‰ and 0.5‰ ( ± 1 sigma) for δ^18^O and δ^2^H, respectively. A detailed description of the analytical procedure used is given in van Geldern and Barth^48^. Following stable isotope analyses, the chloride concentration of the samples was determined by titration with silver nitrate using a METROHM auto-titrator. IAPSO standard seawater (Cl = 19.376 g/kg or as specified on the bottle; the sum of chloride and bromide is 559 mM) was used to calibrate the results and the precision is estimated to be 0.3% based on repeated measurements of IAPSO seawater. Salinity was calculated by assuming all chloride was associated with Na.

### Uncertainty estimation

Uncertainties in δ^18^Osw and SST are estimated by propagating errors introduced by the δ^18^O and Mg/Ca measurements, and by the calibration equations for both temperature and the δ^18^Osw. Uncertainties in SST and δ^18^Osw are on average ~1 °C and ~0.3‰, respectively. For the SST and δ^18^Osw estimates, the following equations from Mohtadi et al.^49^ were used to propagate the errors.

### SST calibration and error propagation

SSTs were calculated by using the multispecies equation of Anand et al.^42^

This is given as follows:$\frac{{\rm Mg}}{{\rm Ca}} = be^{aT}$, where, *b* = 0.38 ± 0.02 mmol/mol; *a* = 0.090 ± 0.003 °C^−1^

Errors associated with SST are estimated by propagating the errors in *a*, *b* and Mg/Ca measurement errors and is given as:

$$\sigma _T^2 = \left( {\frac{{\partial T}}{{\partial a}}\sigma _a} \right)^2 + \left( {\frac{{\partial T}}{{\partial b}}\sigma _b} \right)^2 + \left( {\frac{{\partial T}}{{\partial {\rm Mg}{\mathrm{/}}{\rm Ca}}}\sigma _{\rm Mg{\mathrm{/}}Ca}} \right)^2$$

where, $$\frac{{\partial T}}{{\partial a}} = - \frac{1}{{a^2}}{\mathrm{ln}}\left( {\frac{{\rm Mg{\mathrm{/}}Ca}}{b}} \right)$$, $$\frac{{\partial T}}{{\partial b}} = - \frac{1}{{ab}}$$, and $$\frac{{\partial T}}{{\partial \rm Mg{\mathrm{/}}\rm Ca}} = \frac{1}{a}\frac{1}{{\rm Mg{\mathrm{/}}Ca}}$$

### δ^18^Osw and error propagation

δ^18^Osw were calculated by using the δ^18^O-paleotemperature equation of Bemis et al.^43^, which is given as follows:

$${{T = a}} + {{b}}\left( {{\mathrm{\delta }}^{{\mathrm{18}}}{\mathrm{O}}_{{\mathrm{calcite}}}-{\mathrm{\delta }}^{{\mathrm{18}}}{\mathrm{O}}_{{\mathrm{seawater}}}} \right),$$

where *a* = 16.5 ± 0.2 °C; *b* = −4.80 ± 0.16 °C and *T* is estimated from Mg/Ca measurements as shown above:

Errors associated with δ^18^Osw are estimated by propagating the errors in *a*, *b*, *T* and δ^18^O measurement errors by assuming no covariance among the errors and is given as:

$${{\sigma _{\delta ^{18}{\rm O_{sw}}}^2 = \left( {\frac{{\partial \delta ^{18}{\rm O_{sw}}}}{{\partial T}}\sigma _T} \right)^2 + \left( {\frac{{\partial \delta ^{18}{\rm O_{sw}}}}{{\partial a}}\sigma _a} \right)^2 + \left( {\frac{{\partial \delta ^{18}{\rm O_{sw}}}}{{\partial b}}\sigma _b} \right)^2 + \left( {\frac{{\partial \delta ^{18}{\rm O_{sw}}}}{{\partial \delta ^{18}{\rm O_{calcite}}}}\sigma _{\delta ^{18}{\rm O_{calcite}}}} \right)^2}}$$

where, $$\frac{{\partial \delta ^{18}{\rm O_{sw}}}}{{\partial T}} = - \frac{1}{b}$$, $$\frac{{\partial \delta ^{18}{\rm O_{sw}}}}{{\partial a}} = \frac{1}{b}$$, $$\frac{{\partial \delta ^{18}{\rm O_{sw}}}}{{\partial b}} = \frac{T}{{b^2}} - \frac{a}{{b^2}}$$, and $$\frac{{\partial \delta ^{18}{\rm O_{sw}}}}{{\partial \delta ^{18}{\rm O_{calcite}}}} = 1$$

## Electronic supplementary material

Supplementary Information

## Data Availability

Data underlying the findings of this study are available here: 10.1594/PANGAEA.894886.

## References

[1] Lutz W, Samir K (2011). Global human capital: integrating education and population. Science.

[2] Webster PJ (1998). Monsoons: processes, predictability, and the prospects for prediction. J. Geophys. Res.: Oceans.

[3] Boos WR, Kuang Z (2010). Dominant control of the South Asian monsoon by orographic insulation versus plateau heating. Nature.

[4] Wang Pin Xian, Wang Bin, Cheng Hai, Fasullo John, Guo ZhengTang, Kiefer Thorsten, Liu ZhengYu (2017). The global monsoon across time scales: Mechanisms and outstanding issues. Earth-Science Reviews.

[5] Wang, B. et al. Rethinking Indian monsoon rainfall prediction in the context of recent global warming. *Nat. Commun.* **6**, 7154 (2015). 10.1038/ncomms8154 PMC4479044 25981180

[6] Mani, N. J., Suhas, E. & Goswami, B. Can global warming make Indian monsoon weather less predictable? *Geophys. Res. Lett.* **36**​, L08811 (2009).

[7] Flato, G. et al. *Climate Change 2013: The Physical Science Basis* (eds Stocker, T. F. et al.) 741–866 (Cambridge University Press, UK, 2013).

[8] Berger A, Li X, Loutre MF (1999). Modelling northern hemisphere ice volume over the last 3Ma. Quat. Sci. Rev..

[9] Caley T (2011). New Arabian Sea records help decipher orbital timing of Indo-Asian monsoon. Earth. Planet. Sci. Lett..

[10] Clemens SC, Prell WL (2003). A 350,000 year summer-monsoon multi-proxy stack from the Owen Ridge, Northern Arabian Sea. Mar. Geol..

[11] Prell W, Curry W (1981). Faunal and isotopic indices of monsoonal upwelling-western arabian sea. Oceanol. Acta.

[12] Clemens S, Prell W, Murray D, Shimmield G, Weedon G (1991). Forcing mechanisms of the Indian Ocean monsoon. Nature.

[13] Bolton CT (2013). A 500,000 year record of Indian summer monsoon dynamics recorded by eastern equatorial Indian Ocean upper water-column structure. Quat. Sci. Rev..

[14] Battisti D, Ding Q, Roe G (2014). Coherent pan‐Asian climatic and isotopic response to orbital forcing of tropical insolation. J. Geophys. Res.: Atmospheres.

[15] Cheng H (2016). The Asian monsoon over the past 640,000 years and ice age terminations. Nature.

[16] Cheng H (2009). Ice age terminations. Science.

[17] Wang Y (2008). Millennial-and orbital-scale changes in the East Asian monsoon over the past 224,000 years. Nature.

[18] Wang B, Wu R, Lau K (2001). Interannual variability of the Asian summer monsoon: contrasts between the Indian and the Western North Pacific-East Asian Monsoons*. J. Clim..

[19] Cai Y (2015). Variability of stalagmite-inferred Indian monsoon precipitation over the past 252,000 y. Proc. Natl. Acad. Sci..

[20] Clemens (2018). Precession-band variance missing from East Asian monsoon runoff. Nat. Commun..

[21] Kudrass H, Hofmann A, Doose H, Emeis K, Erlenkeuser H (2001). Modulation and amplification of climatic changes in the Northern Hemisphere by the Indian summer monsoon during the past 80 ky. Geology.

[22] Pant V (2015). Observed interannual variability of near‐surface salinity in the Bay of Bengal. J. Geophys. Res.: Oceans.

[23] Sengupta D, Bharath Raj G, Ravichandran M, Sree Lekha J, Papa F (2016). Near‐surface salinity and stratification in the north Bay of Bengal from moored observations. Geophys. Res. Lett..

[24] Girishkumar M, Ravichandran M, Han W (2013). Observed intraseasonal thermocline variability in the Bay of Bengal. J. Geophys. Res.: Oceans.

[25] Rao, R. & Sivakumar, R. Seasonal variability of sea surface salinity and salt budget of the mixed layer of the north Indian Ocean. *J. Geophys. Res. Oceans* **108**, 3009 (2003).

[26] Guptha M, Curry W, Ittekkot V, Muralinath A (1997). Seasonal variation in the flux of planktic Foraminifera; sediment trap results from the Bay of Bengal, northern Indian Ocean. J. Foraminifer. Res..

[27] Collett, T. S. et al. Indian continental margin gas hydrate prospects: results of the Indian National Gas Hydrate Program (NGHP) expedition 01. In *Proc. of the 6th International Conference on Gas Hydrates* (Univeristy of British Columbia, 2008).

[28] Gebregiorgis D (2016). South Asian summer monsoon variability during the last∼ 54 kyrs inferred from surface water salinity and river run off proxies. Quat. Sci. Rev..

[29] Sijinkumar A (2016). δ 18 O and salinity variability from the last glacial maximum to recent in the Bay of Bengal and Andaman Sea. Quat. Sci. Rev..

[30] Berger, A. & Loutre, M. F. Insolation values for the climate of the last 10 million of years. *Quat. Sci. Rev.* **10**, 297–317 (1991).

[31] Wang Y (2008). Millennial-and orbital-scale changes in the East Asian monsoon over the past 224,000 years. Nature.

[32] Mantsis DF (2014). The response of large-scale circulation to obliquity-induced changes in meridional heating gradients. J. Clim..

[33] Webster PJ, Yang S (1992). Monsoon and ENSO: selectively interactive systems. Q. J. R. Meteorol. Soc..

[34] Kumar KK (2011). The once and future pulse of Indian monsoonal climate. Clim. Dyn..

[35] Lisiecki Lorraine E., Raymo Maureen E. (2005). A Pliocene-Pleistocene stack of 57 globally distributed benthic δ18O records. Paleoceanography.

[36] Paillard D, Labeyrie L, Yiou P (1996). Macintosh program performs time‐series analysis. Eos, Trans. Am. Geophys. Union.

[37] Shackleton NJ (1984). Oxygen isotope calibration of the onset of ice-rafting and history of glaciation in the North Atlantic region. Nature.

[38] Ali S (2015). South Asian monsoon history over the past 60 kyr recorded by radiogenic isotopes and clay mineral assemblages in the Andaman Sea. Geochem., Geophys., Geosystems.

[39] Southon J, Kashgarian M, Fontugne M, Metivier B, Yim WW (2002). Marine reservoir corrections for the Indian Ocean and Southeast Asia. Radiocarbon.

[40] Greaves, M. et al. Interlaboratory comparison study of calibration standards for foraminiferal Mg/Ca thermometry. *Geochem. Geophys.* **9**, Q08010 (2008).

[41] Barker S., Greaves M., Elderfield H. (2003). A study of cleaning procedures used for foraminiferal Mg/Ca paleothermometry. Geochemistry, Geophysics, Geosystems.

[42] Anand Pallavi, Elderfield Henry, Conte Maureen H. (2003). Calibration of Mg/Ca thermometry in planktonic foraminifera from a sediment trap time series. Paleoceanography.

[43] Bemis E, Spero HJ, Bijma J, Lea DW (1998). Reevaluation of the oxygen isotopic composition of planktonic foraminifera: Experimental results and revised paleotemperature equations. Paleoceanography.

[44] Rohling E (2014). Sea-level and deep-sea-temperature variability over the past 5.3 million years. Nature.

[45] Waelbroeck C (2002). Sea-level and deep water temperature changes derived from benthic foraminifera isotopic records. Quat. Sci. Rev..

[46] Schulz M, Mudelsee M (2002). REDFIT: estimating red-noise spectra directly from unevenly spaced paleoclimatic time series. Comput. Geosci..

[47] Howell, P. ARAND time series and spectral analysis package for the Macintosh, Brown University. In *IGBP PAGES/World Data Center for Paleoclimatology Data Contribution Series* Vol. 44 (World Data Center for Paleoclimatology, 2001).

[48] Geldern R, Barth JA (2012). Optimization of instrument setup and post‐run corrections for oxygen and hydrogen stable isotope measurements of water by isotope ratio infrared spectroscopy (IRIS). Limnol. Oceanogr.: Methods.

[49] Mohtadi M (2014). North Atlantic forcing of tropical Indian Ocean climate. Nature.

[50] Levitus (2013). The world ocean database. Data Sci. J..

[51] Achyuthan H (2013). Stable isotopes and salinity in the surface waters of the Bay of Bengal: implications for water dynamics and palaeoclimate. Mar. Chem..

[52] Laskar J, Joutel F, Boudin F (1993). Orbital, precessional, and insolation quantities for the Earth from-20 Myr to+10 Myr. Astron. Astrophys..

[53] Berger A, Loutre MF (1997). Intertropical latitudes and precessional and half-precessional cycles. Science.

