# Supplementary material for: Southern Hemisphere forcing of South Asian monsoon precipitation over the past ~1 million years
Source: Nat Commun. 2018 Nov 8;9:4702. doi: 10.1038/s41467-018-07076-2 (PMC6224551; doi:10.1038/s41467-018-07076-2)
Supplement: Supplementary file 1 — Supplementary Information [file 41467_2018_7076_MOESM1_ESM.pdf]

Southern Hemisphere forcing of South Asian monsoon precipitation over the past 1 million years

D. Gebregiorgis et al.

Supplementary Note 1: Modern oceanography of the Bay of Bengal

The Andaman Sea and Bay of Bengal form a unique semi-enclosed tropical basin in the northeastern Indian Ocean that experiences a dominant seasonal surface wind reversal during boreal summer (June–September) and winter (November–February) seasons. Modern day upper ocean variability in the Andaman Sea and Bay of Bengal is characterised by a semi-annual cycle of mixed layer and thermocline depth variations and is intrinsic to the seasonally reversing monsoonal circulation[1,2]. The net annual surface water exchange (i.e. precipitation plus runoff minus evaporation) is primarily driven by intense freshening of the surface ocean during the summer monsoon season and leads to strong stratification of the upper ocean, which persists year round[3]. This freshwater forcing also exerts significant influence on SST patterns and SSS distribution in the tropical belt[4] and instigates a large reduction in SSS. The mean SSS in the Andaman Sea ranges between 28 and 33‰ (Ref 5). Mean SST is 29°C and homogenous throughout the mixed layer and the top of the thermocline, which is generally situated at a depth of ~50 m (Ref 6). Although near-surface SSS changes in the Bay of Bengal are dominated by the freshwater fluxes associated with the summer monsoon, it has also been reported that vertical mixing plays a significant role in controlling near-surface salinity in this region[7,8,9]. The vertical movement of the thermocline in particular is known to play an important role in driving chlorophyll-*a* concentration and biological activities in the upper ocean by modulating the advection of nutrient rich thermocline waters[10]. A sediment trap study conducted along a transect in the Bay of Bengal showed that the abundance of mixed layer dwelling planktonic foraminifera *G. sacculifer* is not biased towards a particular season[11]. This is also the case with the thermocline dwelling *N. dutertrei*, which is present throughout the year with a minor peak in abundance during the summer monsoon[11]. Thus, seasonal biases are not anticipated in these species and long-term changes in these records represent ‘year round’ surface and thermocline conditions. We assume that the living depths of the two species likely remained similar to modern day observations[12,13].

A recent comprehensive study examining the seasonality of SST and SSS in the Bay of Bengal based on observations from a mooring in the Bay of Bengal demonstrate that SSS changes in the Bay of Bengal to large extent are driven by river discharge from the Ganges-Brahmaputra and Irrawaddy rivers during the summer monsoon season[14]. The study[14] also notes that this co-variability occurs with a significant time lag (Supplementary Figure 7) and low surface salinity changes are often observed during the following winter monsoon season. Thus, the δ^{18}Osw record is interpreted in the context of monsoon variability proportional to salinity changes in the Andaman Sea. This relationship is also clearly supported by data from the last deglaciation (Supplementary Figure 8) and by the prevalence of a remarkably consistent patterns in the *G. sacculifer* and *N. dutertrei* based δ^{18}Osw records which approximate mixed layer and thermocline conditions at the base of the boundary layer (Supplementary Figure 9). Physical processes such as wind-stress curl via Ekman pumping, geostrophic flow, and the radiation of Rossby waves from poleward propagating coastal Kelvin waves influence the seasonal thermocline[15]. The vertical movement of the thermocline also plays an important role in driving chlorophyll-*a* concentration and biological activities in the upper ocean by modulating the advection of nutrient rich thermocline waters[10]. Thermocline depth variability also exerts considerable influence on SST by transferring colder thermocline waters into the upper ocean through wind-induced upwelling, entrainment and mixing[1]. Recent work has shown *N. dutertrei* to feed on marine snow[16] and particulate material both terrigenous and autochthonous would accumulate preferentially at the base of the boundary layer suggesting this is the likely habitat for *N. dutertrei* in the BoB and Andaman Sea. This demonstrates that the δ^{18}O records (Supplementary Figure 10) have primarily been driven by surface ocean rather than intermediate water variability.

Supplementary Note 2: Salinity influence on Mg/Ca temperatures

The oxygen isotopic composition (δ^{18}O) and Mg/Ca ratios of planktic foraminifera tests are among the most commonly applied proxies for reconstructing past

ocean temperature and δ^{18}Osw. Recent studies, however, have highlighted the potential influence of salinity on Mg/Ca ratios[17,18,19], although any significant influence of salinity on Mg/Ca is generally driven by higher Mg/Ca ratios in regions with salinities above 35 psu. For example, the new data for Bay of Bengal sediment trap samples from Gray et al.[17] demonstrate the absence of a salinity influence on Mg/Ca over the Bay (see figure 3b in Ref 17) and the salinity effect is driven by the Arabian Sea samples. Nonetheless, reconstructed salinity (indirectly inferred from the $\delta^{18}O_{SW}$ record) was higher in the Andaman Sea during glacials, and therefore, we have carefully checked for any potential salinity influence on the Mg/Ca-SST record using the PSU solver program[20]. We find that salinity did not significantly affect the Mg/Ca and, consequently, also not the resulting $\delta^{18}O_{SW}$ records (Supplementary Figure 11).

In addition, to test if the δ^{18}Osw signal is sensitive to potential artefacts arising from sea level correction due to the fact that the periodicities of mechanisms driving both temperature and sea level are the same (glacial-interglacial cyclicity), we have performed spectral analysis on the SST and raw δ^{18}O records (Supplementary Figure 12). Reconstructed SST and raw δ^{18}O records are dominated by pronounced ~100 kyr eccentricity, 41 kyr obliquity and ~23 kyr precession cycles (>90% CI) in line with δ^{18}Osw. We have also compared the temporal evolution of IV corrected and IV uncorrected $\delta^{18}O_{SW}$ records (Supplementary Figure 13) and estimate the orbital phasing of the two (Supplementary Figure 14). The IV corrected and IV uncorrected $\delta^{18}O_{SW}$ records are significantly coherent with respect to ETP[21] in the precession band and show a consistent time lag of ~8-9 kyrs (-148°/360° * 23 kyrs) behind NH insolation maxima. On the other hand the IV uncorrected $\delta^{18}O_{SW}$ lags the IV corrected timeseries by ~11 kyrs and phases closer to the minimum sea level (maximum IV) pointing to this frequency outcompeting the monsoon signal.

Figure captions for supplementary material

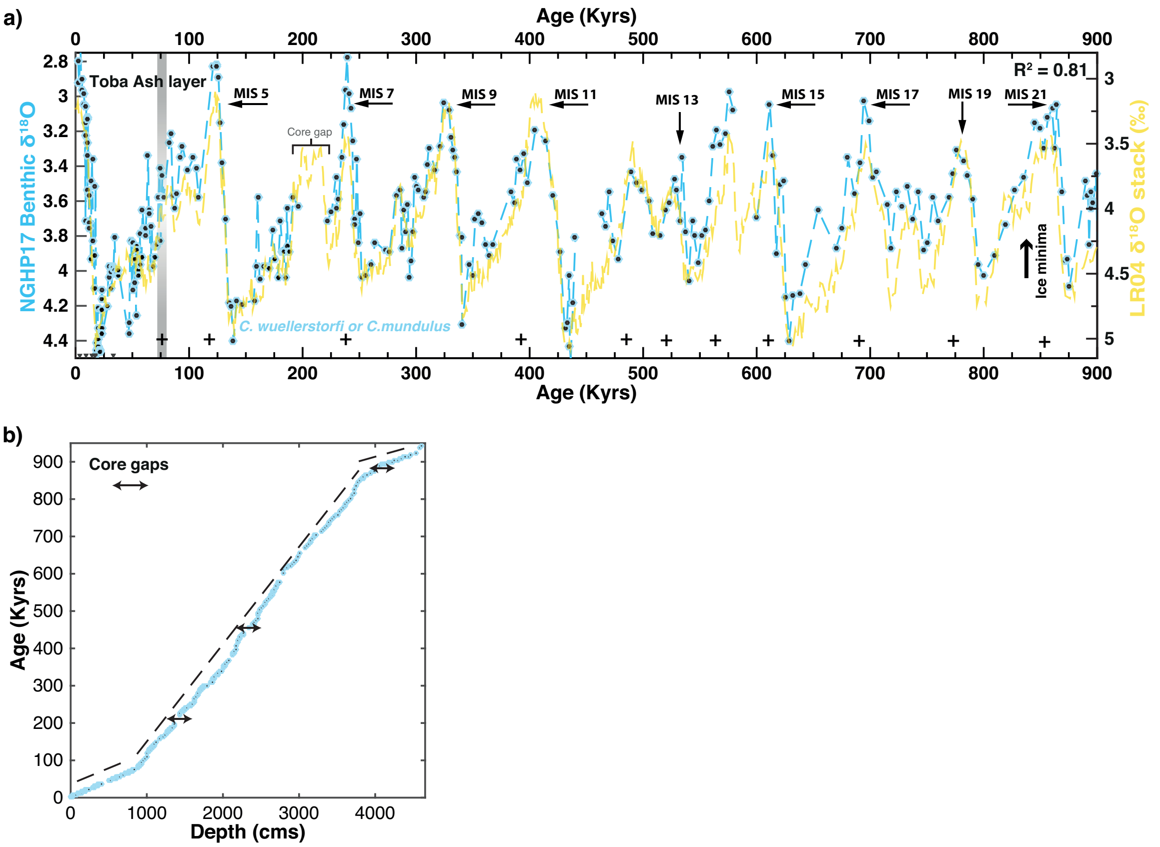

Supplementary Figure 1. a) Correlation of the Site NGHP17 benthic (C. wuellerstorfi /C. mundulus) $\delta^{18}O$ record (yellow) and the LR04 benthic stack[22]. Major Tie points are shown by crosses. b) Age depth model for NGHP 17 indicating a near linear sedimentation rate.

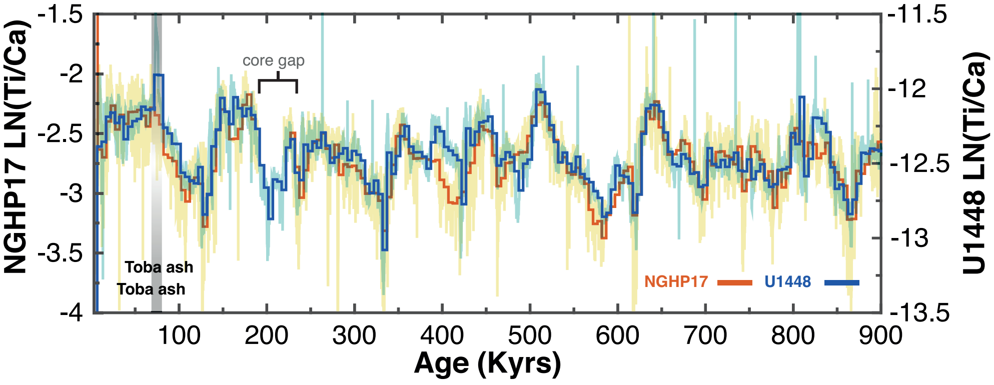

Supplementary Figure 2. Crosshole comparison of XRF Ti/Ca records of two Andaman Sea Sites (NGHP17 and U1448) to establish the length of gaps between cores that result from the drilling process.

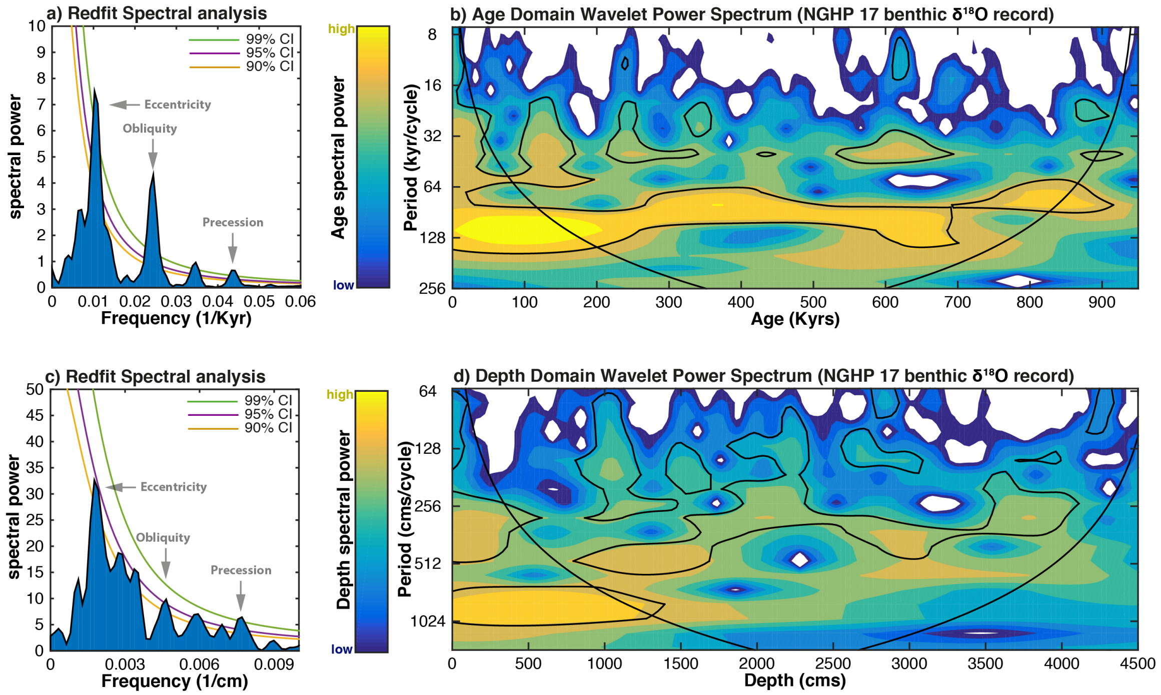

Supplementary Figure 3. a) Redfit spectral analysis[23] of LR04 tuned Site 17 benthic isotope stratigraphy showing strong periodicity at frequencies corresponding to the three main orbital periods (95% CI). b) Continuous wavelet transform of NGHP 17 benthic isotope record. c) Redfit spectral analysis[23] of NGHP 17 benthic record on depth domain showing strong power spectra at 0.002, 0.005 and 0.007 m/cycle (95% CI). d) Continuous wavelet transform of NGHP 17 benthic record in depth domain. Black line contours in b) and d) represent 90% confidence level. The evolutionary wavelet spectrum is computed using the Matlab codes of Torrence and Compo[24] available at (paos.colorado.edu/research/wavelets/).

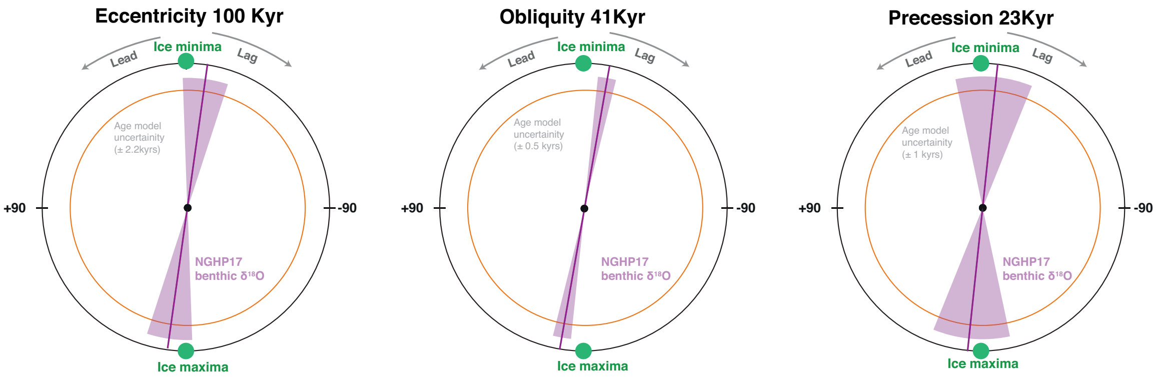

Supplementary Figure 4. Phase wheels summarizsng age model uncertainties with respect to LR04. Ice minima and maxima defined based on the LR04 benthic stack[22].

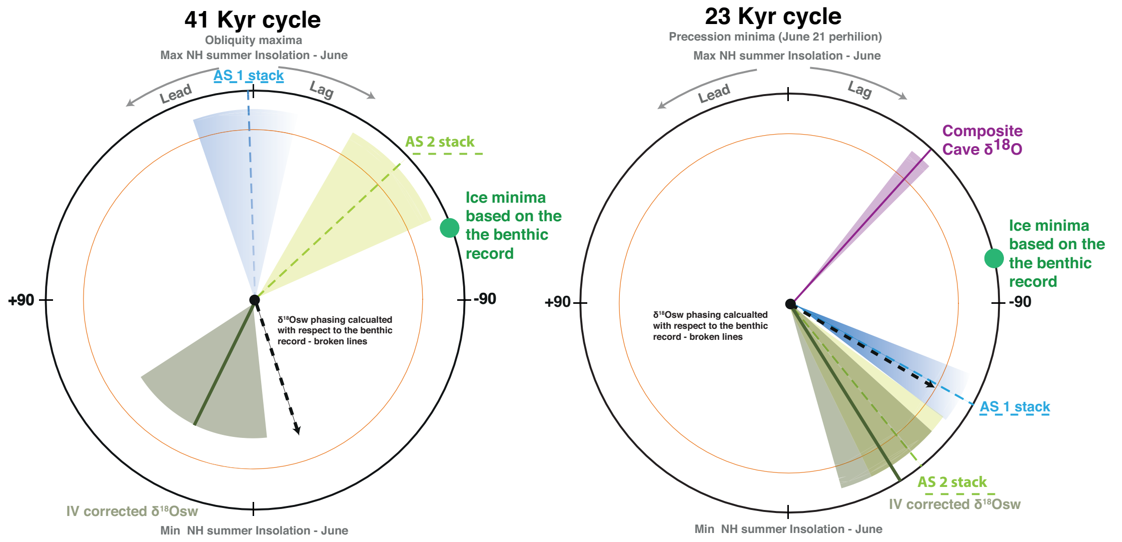

Supplementary Figure 5. Phase wheels summarising Asian monsoon response to orbital insolation forcing at the obliquity (41kyr) and precession (23 kyr) periods during early to late Pleistocene as in Fig. 2 with precession related maximum and minimum insolation based on the NGHP17 benthic record.

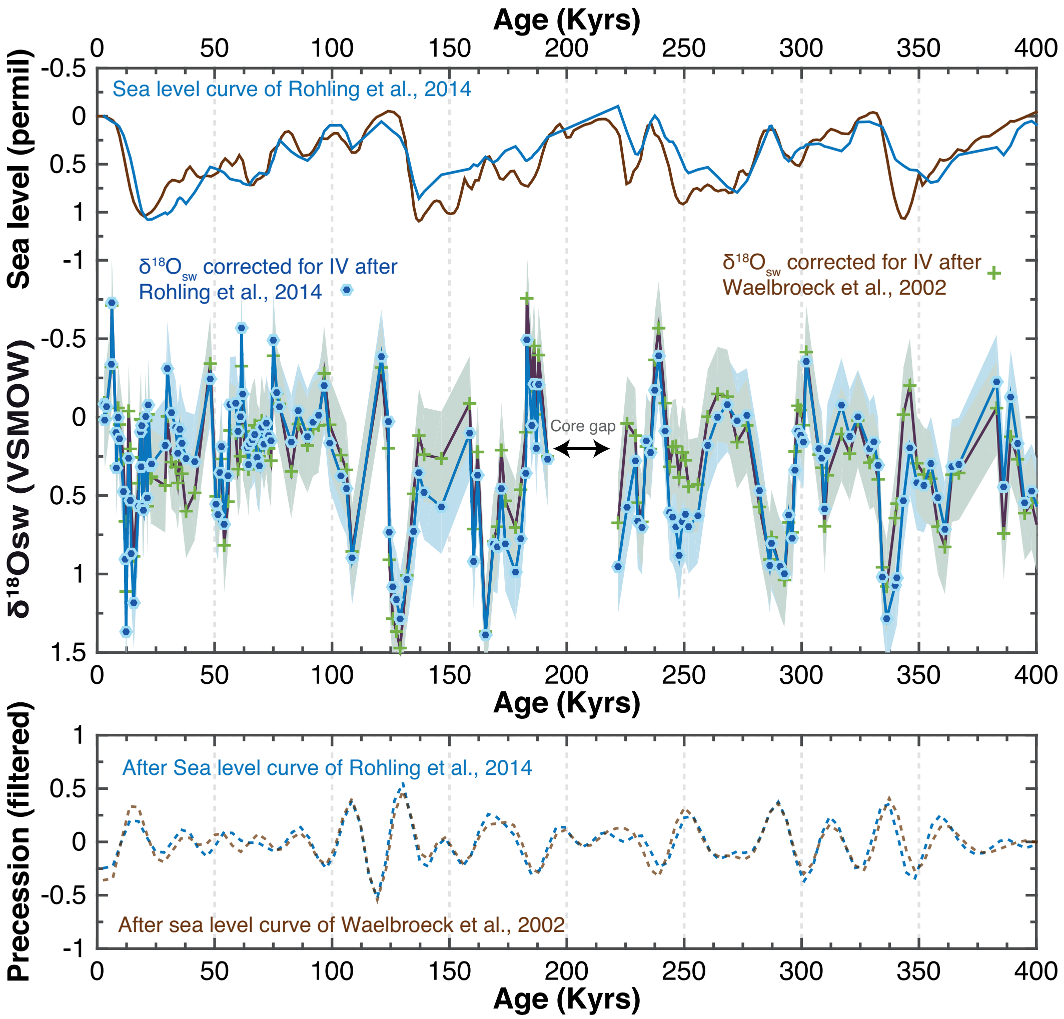

Supplementary Figure 6. Comparison of the sea level curve from Rohling et al.[25] used to correct for ice volume with a sea level curve from Waelbroeck et al.[26] and the NGHP17 benthic data.

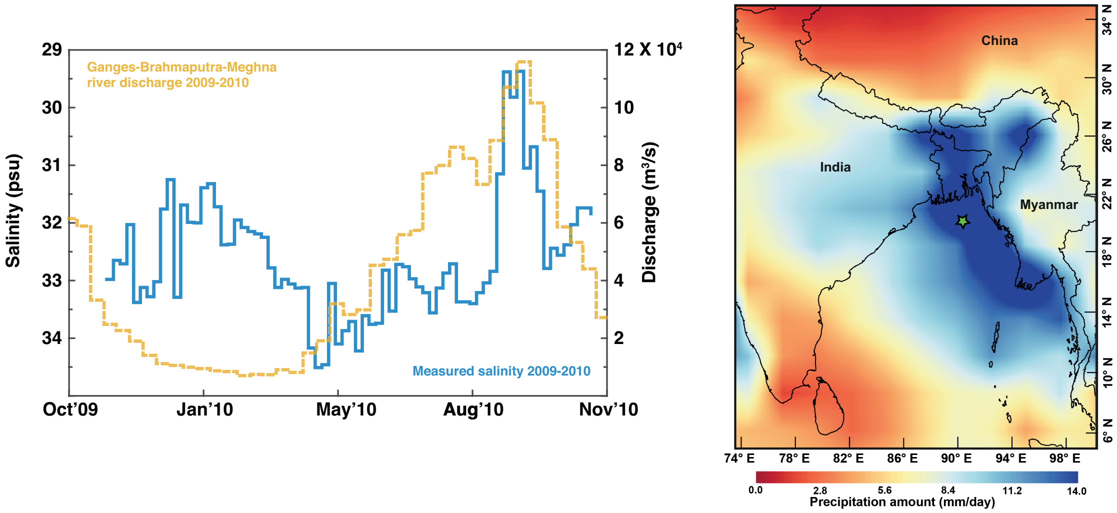

Supplementary Figure 7. Comparison of Bay of Bengal salinity data from moored observations[14] and Ganges-Brahmaputra-Meghna river discharge (km^3/day) based on satellite observations[27,28]

between 2009-2010. Also shown is mean summer monsoon precipitation in the Bay of Bengal for the period 1979-2015.

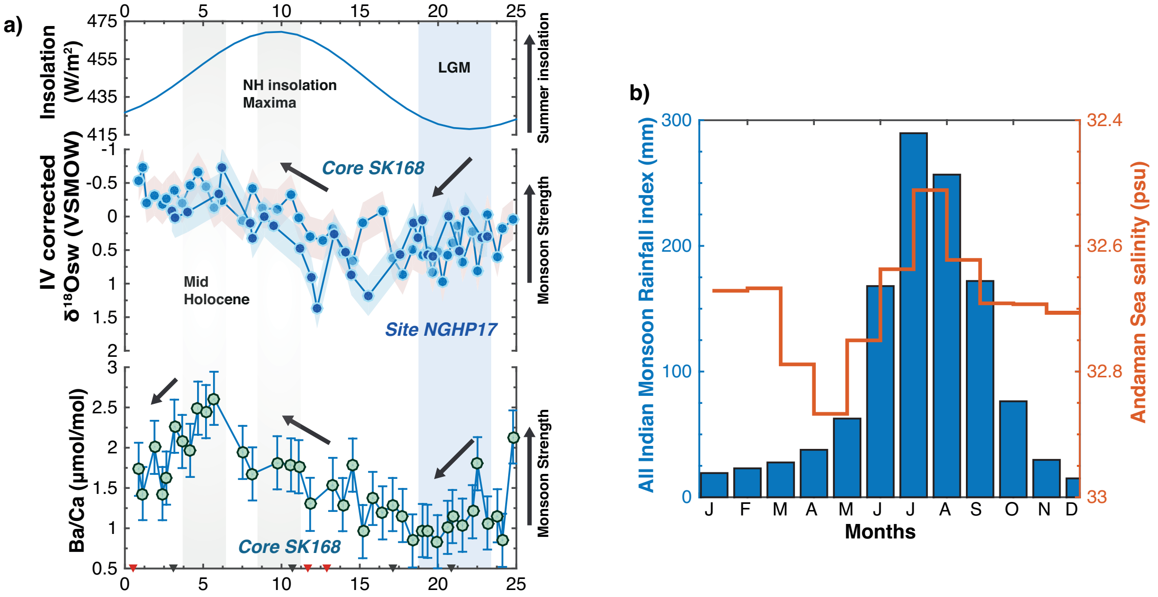

Supplementary Figure 8. a) Deglacial multi-proxy records of the monsoon from core SSK168 (Ref 29,30) and NGHP17 (see Fig. 1) in relation to changes in insolation. b) Seasonal monsoon precipitation inferred from All Indian Monsoon Rainfall Index (AIMRI) and Andaman Sea salinity variations extracted from SODA for the year 1959-2013. Inverted triangles in red and black show ^{14}C-AMS dates used to construct the age models for cores NGHP17 and SSK168 respectively.

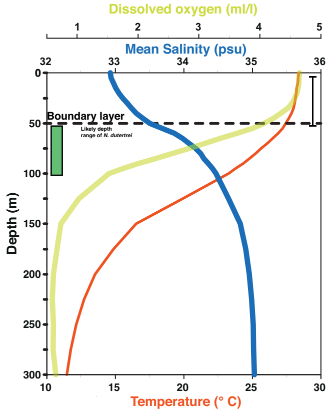

Supplementary Figure 9. Modern depth profiles of temperature, salinity and dissolved oxygen from World Ocean Atlas 2013 (Ref 5). Green bar shows depth range of deep chlorophyll maximum (DCM) during May-June 1996 (Ref 31).

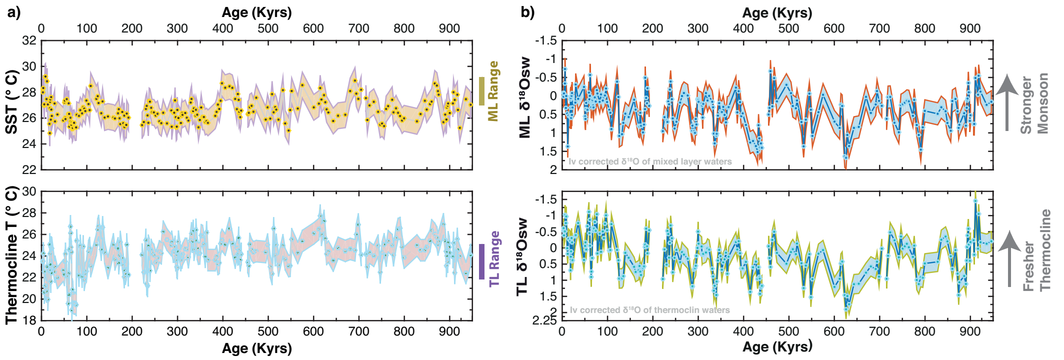

Supplementary Figure 10. a) Reconstructed SST and thermocline temperatures (calculated based on Mg/Ca analysis of N. dutertrei) b) Ice volume corrected δ^{18}Osw and thermocline δ^{18}Osw (VPDP Scale). Envelops in a and b show propagated errors. SSTs were calculated by using the multispecies equation of Anand et al.[32]. Seawater δ^{18}O ($\delta^{18}O_{SW}$) was calculated using the δ^{18}O – temperature calibration of Bemis et al.[33].

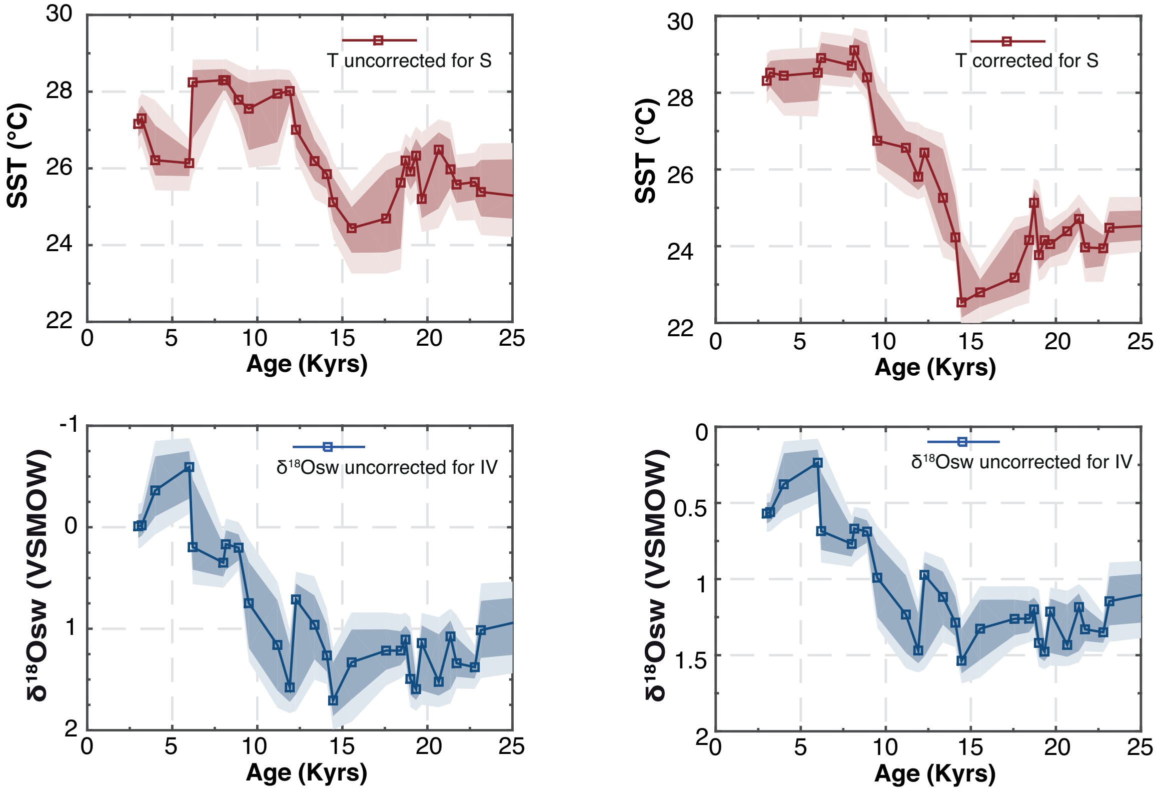

Supplementary Figure 11. Estimating the relative influence of salinity on the Mg/Ca ratios based on the Mg/Ca-SST-SSS equation of Kisakürek et al.[34] using the PSU solver program[20]. Envelopes denote the 68% and 95% uncertainty range due to analytical, and sampling errors.

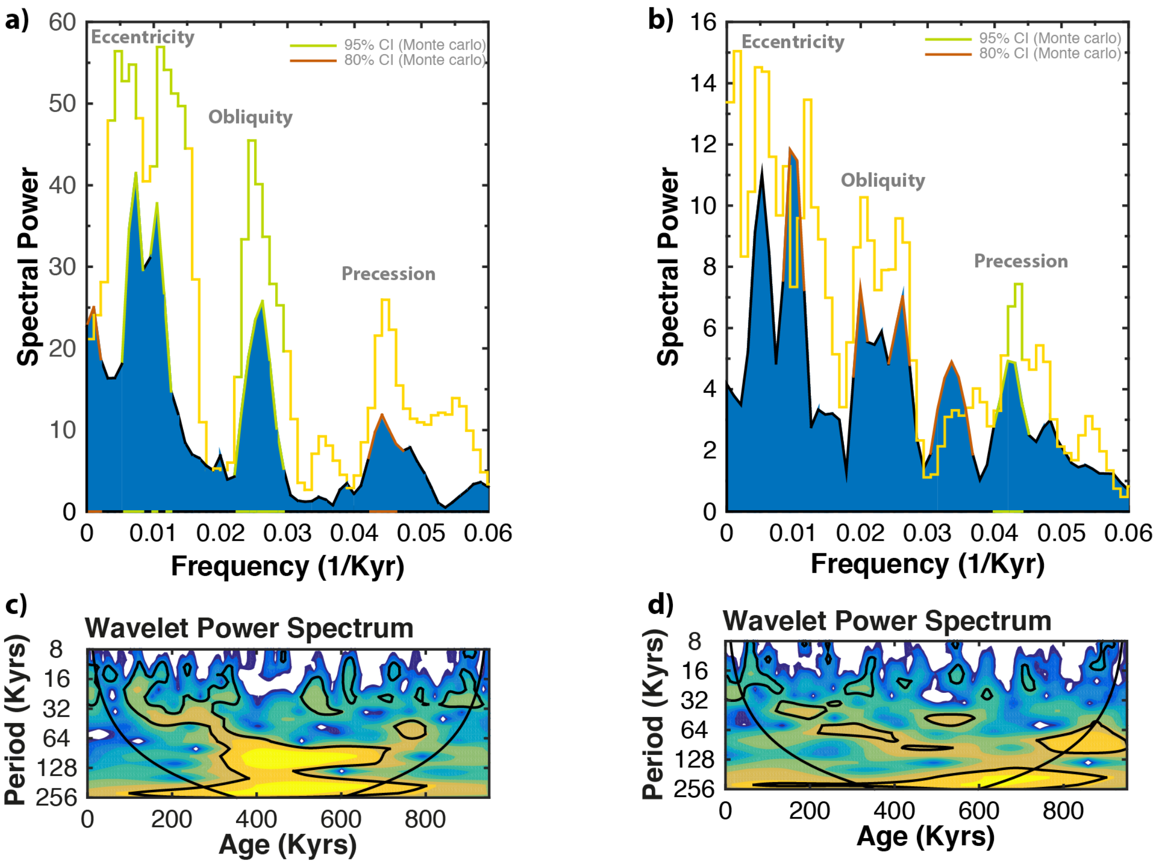

Supplementary Figure 12. Redfit Spectral analysis[23] of a) SST (blue) and thermocline temperature (yellow) records. b) Redfit spectral analysis[23] of ice volume corrected δ^{18}Osw (blue) and thermocline δ^{18}Osw (yellow) records. Significant spectral powers at the 95% CI and 80% CI are shown in light green and brown respectively. c) Continuous wavelet transform[24] of Ice volume corrected δ^{18}Osw. d) Continuous wavelet transform[24] of Ice volume corrected thermocline δ^{18}Osw. Black contours in c) and d) represent 80% confidence level.

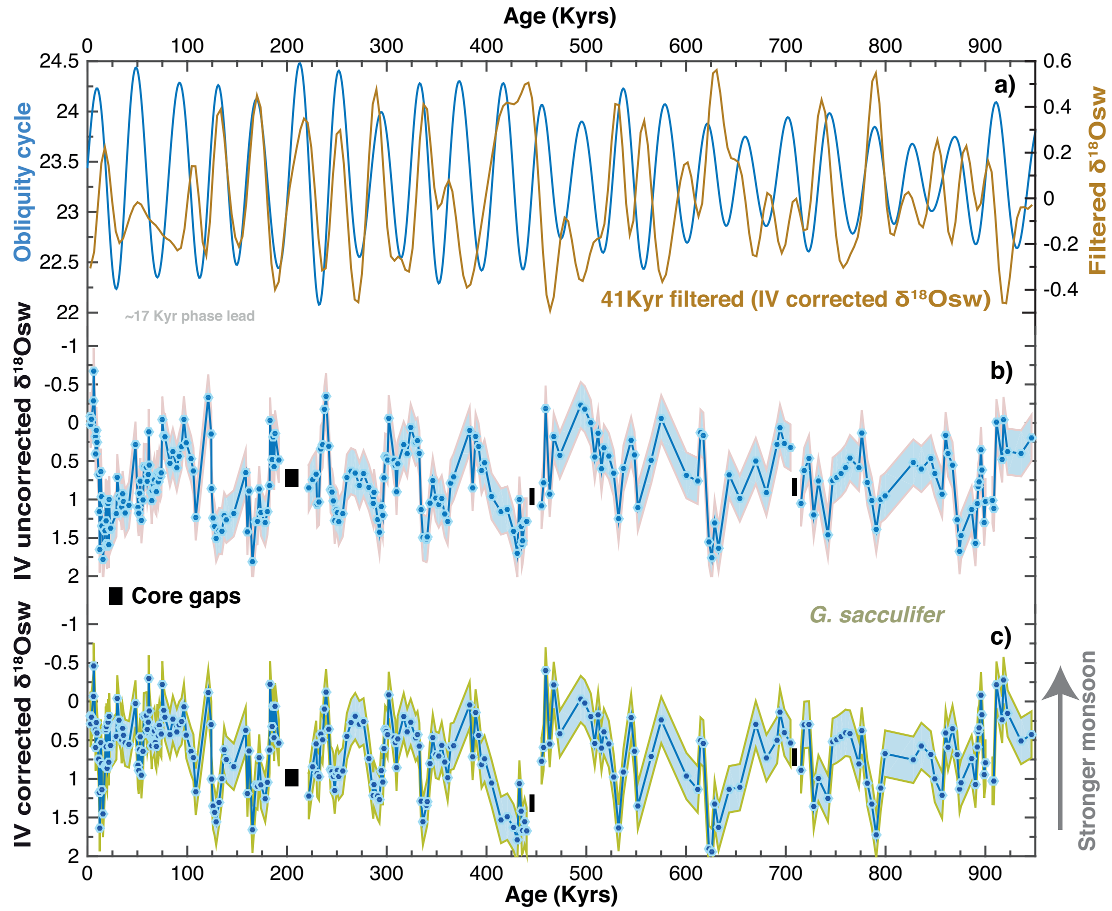

Supplementary Figure 13. a) Comparison of 41 kyr obliquity[21] (blue) and 41 kyrs filtered IV corrected δ^{18}Osw. b) Time series of reconstructed δ^{18}Osw uncorrected for ice volume. c) Ice volume corrected δ^{18}Osw. Envelopes in b and c show 1σ-error (see methods).

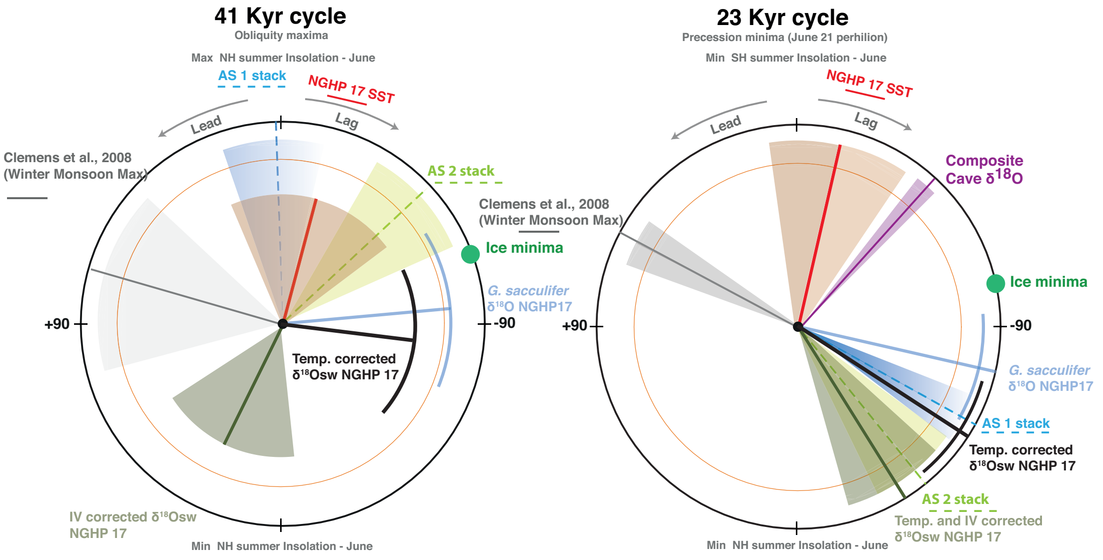

Supplementary Figure 14. Phase wheels summarising Asian monsoon response to orbital insolation forcing at the obliquity (41 kyr) and precession (23 kyr) periods during the late Pleistocene as in Fig. 6 and also showing the phase of reconstructed δ^{18}Osw uncorrected for ice

volume (black line). 95% and 80 confidence intervals for coherency are 0.79 and 0.64 with bandwidth 0.00423175. The interpolated time interval is 3.5 kyrs and covers the period of time from 3 to 951 kyrs.

Supplementary references

1 Girishkumar, M., Ravichandran, M. & Han, W. Observed intraseasonal thermocline variability in the Bay of Bengal. *Journal of Geophysical Research: Oceans* **118**, 3336-3349 (2013).

2 Rao, R. & Sivakumar, R. Seasonal variability of sea surface salinity and salt budget of the mixed layer of the north Indian Ocean. *Journal of Geophysical Research: Oceans* **108** (2003).

3 Prasanna Kumar, S. *et al.* Why is the Bay of Bengal less productive during summer monsoon compared to the Arabian Sea? *Geophysical Research Letters* **29** (2002).

4 Seo, H., Xie, S.-P., Murtugudde, R., Jochum, M. & Miller, A. J. Seasonal effects of Indian Ocean freshwater forcing in a regional coupled model*. *Journal of Climate* **22**, 6577-6596 (2009).

5 Zweng, M. M. *et al.* World ocean atlas 2013. Volume 2, Salinity. (2013).

6 Riser, S., Nystuen, J. & Rogers, A. Monsoon effects in the Bay of Bengal inferred from profiling float-based measurements of wind speed and rainfall. *Limnology and Oceanography* **53**, 2080-2093 (2008).

7 Vinayachandran, P. *et al.* A summer monsoon pump to keep the Bay of Bengal salty. *Geophysical Research Letters* **40**, 1777-1782 (2013).

8 Benshila, R. *et al.* The upper Bay of Bengal salinity structure in a high-resolution model. *Ocean Modelling* **74**, 36-52 (2014).

9 Akhil, V. *et al.* A modeling study of processes controlling the Bay of Bengal sea surface salinity interannual variability. *Journal of Geophysical Research: Oceans* **121**, 8471-8495 (2016).

10 Uz, B. M., Yoder, J. A. & Osychny, V. Pumping of nutrients to ocean surface waters by the action of propagating planetary waves. *Nature* **409**, 597-600 (2001).

11 Guptha, M., Curry, W., Ittekkot, V. & Muralinath, A. Seasonal variation in the flux of planktic Foraminifera; sediment trap results from the Bay of Bengal, northern Indian Ocean. *The Journal of Foraminiferal Research* **27**, 5-19 (1997).

12 Cullen, J. L. Microfossil evidence for changing salinity patterns in the Bay of Bengal over the last 20 000 years. *Palaeogeography, Palaeoclimatology, Palaeoecology* **35**, 315-356 (1981).

13 Field, D. B. Variability in vertical distributions of planktonic foraminifera in the California Current: Relationships to vertical ocean structure. *Paleoceanography* **19** (2004).

14 Sengupta, D., Bharath Raj, G., Ravichandran, M., Sree Lekha, J. & Papa, F. Near-surface salinity and stratification in the north Bay of Bengal from moored observations. *Geophysical Research Letters* **43**, 4448-4456 (2016).

15 Yu, L. Variability of the depth of the 20 C isotherm along 6 N in the Bay of Bengal: Its response to remote and local forcing and its relation to satellite SSH variability. *Deep Sea Research Part II: Topical Studies in Oceanography* **50**, 2285-2304 (2003).

16 Fehrenbacher, J. S. *et al.* Ba/Ca ratios in the non-spinose planktic foraminifer Neogloboquadrina dutertrei: Evidence for an organic aggregate microhabitat. *Geochimica et Cosmochimica Acta* (2018).

17 Gray, W. R. *et al.* The effects of temperature, salinity, and the carbonate system on Mg/Ca in Globigerinoides ruber (white): A global sediment trap calibration. *Earth and Planetary Science Letters* **482**, 607-620 (2018).

18 Mathien-Blard, E. & Bassinot, F. Salinity bias on the foraminifera Mg/Ca thermometry: Correction procedure and implications for past ocean hydrographic reconstructions. *Geochemistry, Geophysics, Geosystems* **10** (2009).

19 Hönisch, B. *et al.* The influence of salinity on Mg/Ca in planktic foraminifers–Evidence from cultures, core-top sediments and complementary δ18O. *Geochimica et Cosmochimica Acta* **121**, 196-213 (2013).

20 Thirumalai, K., Quinn, T. M. & Marino, G. Constraining past seawater δ18O and temperature records developed from foraminiferal geochemistry. *Paleoceanography* **31**, 1409-1422 (2016).

21 Berger, A. Parameters of the Earths orbit for the last 5 Million years in 1 kyr resolution. *PANGAEA, doi* **10** (1999).

22 Lisiecki, L. E. & Raymo, M. E. A Pliocene-Pleistocene stack of 57 globally distributed benthic $\delta^{18}O$ records. *Paleoceanography* **20** (2005).

23 Schulz, M. & Mudelsee, M. REDFIT: estimating red-noise spectra directly from unevenly spaced paleoclimatic time series. *Computers & Geosciences* **28**, 421-426 (2002).

24 Torrence, C. & Compo, G. P. A practical guide to wavelet analysis. *Bulletin of the American Meteorological society* **79**, 61-78 (1998).

25 Rohling, E. *et al.* Sea-level and deep-sea-temperature variability over the past 5.3 million years. *Nature* **508**, 477-482 (2014).

26 Waelbroeck, C. et al. Sea-level and deep water temperature changes derived from benthic foraminifera isotopic records. Quaternary Science Reviews 21, 295-305 (2002).

27 Papa, F., Durand, F., Rossow, W., Rahman, A. & Bala, S. Seasonal and Interannual Variations of the Ganges-Brahmaputra River Discharge, 1993-2008 from satellite altimeters. J. Geophys. Res. 115, C12013 (2010)..

28 Papa, F. et al. Ganga-Brahmaputra river discharge from Jason-2 radar altimetry: an update to the long-term satellite-derived estimates of continental freshwater forcing flux into the Bay of Bengal. Journal of Geophysical Research: Oceans 117 (2012).

29 Gebregiorgis, D. *et al.* South Asian summer monsoon variability during the last~ 54 kyrs inferred from surface water salinity and river run off proxies. *Quaternary Science Reviews* **138**, 6-15 (2016).

30 Sijinkumar, A. *et al.* δ 18 O and salinity variability from the Last Glacial Maximum to Recent in the Bay of Bengal and Andaman Sea. *Quaternary Science Reviews* **135**, 79-91 (2016).

31 Murty, V. *et al.* Effect of vertical stability and circulation on the depth of the chlorophyll maximum in the Bay of Bengal during May–June, 1996. *Deep Sea Research Part I: Oceanographic Research Papers* **47**, 859-873 (2000).

32 Anand, P., Elderfield, H. & Conte, M. H. Calibration of Mg/Ca thermometry in planktonic foraminifera from a sediment trap time series. *Paleoceanography* **18** (2003).

33 Bemis, E., Spero, H. J., Bijma, J. & Lea, D. W. Reevaluation of the oxygen isotopic composition of planktonic foraminifera: Experimental results and revised paleotemperature equations. *Paleoceanography* **13**, 150-160 (1998).

34 Kisakürek, B., Eisenhauer, A., Böhm, F., Garbe-Schönberg, D. & Erez, J. Controls on shell Mg/Ca and Sr/Ca in cultured planktonic foraminiferan, Globigerinoides ruber (white). Earth and Planetary Science Letters **273**, 260-269 (2008).
